# Optical ammonia sensors based on fluorescent aza-BODIPY dyes— a flexible toolbox

**DOI:** 10.1007/s00216-020-02891-3

**Published:** 2020-08-24

**Authors:** Maximilian Maierhofer, Veronika Rieger, Torsten Mayr

**Affiliations:** Institute of Analytical Chemistry and Food Chemistry, Graz University of Technology, Stremayrgasse 9/2, 8010 Graz, Austria

**Keywords:** pH indicator, Phase fluorimetry, Dual-lifetime referencing, Bioprocessing, Online monitoring, Chemical sensor

## Abstract

We present three types of optical ammonia sensors suitable for environmental, bioprocess, and reaction monitoring. A respective fluorescent BF_2_-chelated tetraarylazadipyrromethene dye (aza-BODIPYs) is physically entrapped in a polyurethane hydrogel (HydroMed D4) forming an emulsion system with vinyl-terminated polydimethylsiloxane (PDMS). The analyte-sensitive layer is covered by a hydrophobic membrane which excludes hydrophilic substances. Three different protection layers are tested, whereby the Teflon and the hydrophobic PES layers outperform a PDMS/TiO_2_ layer. Response times within their dynamic range of 15 s can be achieved, whereas the PDMS/TiO_2_-covered sensor requires at least 390 s. The three sensors entail the following concentration areas: first sensor 3 μg L^−1^–3 mg L^−1^ (LOD 0.23 μg L^−1^), second sensor 0.1–30 mg L^−1^ (LOD 28 μg L^−1^), and third sensor 3 mg L^−1^–1 g L^−1^ (LOD 0.51 mg L^−1^). Readout is performed with a commercially available phase fluorimeter combined with optical fibers. Dual-lifetime referencing (DLR) is used as referencing method and Egyptian blue acts as an inert reference material. No cross-sensitivity to pH changes can be detected.

**Electronic supplementary material:**

The online version of this article (10.1007/s00216-020-02891-3) contains supplementary material, which is available to authorized users.

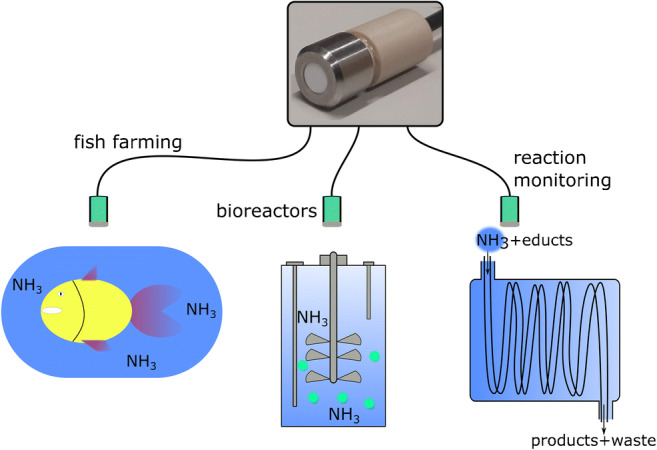

## Introduction

Ammonia is an ubiquitous compound. It is used by humans in industrial fertilizers and refrigerants. Furthermore, it is produced by animals as an excretion product as well as a product in amino acid degradation and waste decomposition [1]. Since there are many origins for ammonia on our planet, it can be found in different habitats, such as river [2] and seawater [3], the atmosphere [4], and soil. As ammonia is toxic for most animals and humans even in trace concentrations, it is an analyte of high interest in environmental monitoring [5]. Due to rising earth population and dealing with the problem of overfishing in the seas, fish farming is gaining more attention. Thereby, ammonia is a crucial parameter which has to be monitored carefully [6]. Even to many microorganisms, ammonia is a toxic compound. Continuous mammalian cell lines (CCLs), which are important hosts for the production of biological pharmaceuticals, generate ammonia during the cell metabolism. This is due to the lack of energy in form of ATP (adenosine-5′-triphosphate) [7, 8]. To close this gap, these cells consume glutamine to produce α-ketoglutarate, which is further degraded in the mitochondria and as a second product poising ammonia [9]. Ammonia inhibits cell growth and further yields to cell death [10].

Many different methods for the determination of dissolved ammonia have been developed. Thereby, IR measurements [11] as well as electrochemical approaches like potentiometric electrodes [12] and conductometric [13], photoacoustic [14, 15] photothermal [16], and amperometric methods [2] are worth mentioning. In analytical routine analysis, colorimetric methods based on Berthelot’s [17, 18] and Nessler’s reaction [19] are well established. However, these methods either consume analyte, convert the analyte chemically into another species, or require expensive and bulky instrumentation. Others need sample preparation making them less suitable for fieldtrips or online monitoring. The colorimetric methods lack a slow chemical reaction kinetic and require trained personal in the sample preparation step. Since we wanted to develop a low-cost sensor which is suitable for online monitoring, we have chosen a different sensing technique: Within the concept of optical sensing, different indicator dye classes suitable for ammonia sensing have been already published. Xanthene dyes [20–24], coumarin derivatives [25–27], Ru(II) polypyridyl dyes [28], and triphenylmethane dyes [29, 30] have been investigated intensively. Most dyes of these classes show poor photostability/long-term stability and are not sensitive enough for trace measurements. This fact makes them unsuitable for environmental monitoring.

We present an optical ammonia sensor based on BF_2_-chelated tetraarylazadipyrromethene dyes (aza-BODIPY). These show high photostability [31], are excitable with low-cost red LEDs (~ 620 nm), and emit in the near infrared region of the spectrum (650–750 nm) [32]. Within this region, autofluorescence of biomolecules is decreased yielding in less background scattering. This decrease makes the sensor more sensitive. Our sensing system relies on an acid–base concept. Here, the analyte in its gaseous form diffuses through a porous, hydrophobic membrane into the sensing layer. It deprotonates the indicator dye inducing fluorescence quenching [33]. With varying p*K*_a_ of the dye’s hydroxyl group, we can tune the sensitivity of our optical ammonia sensor offering different applications in the broad field of analytical ammonia detection. The readout is performed via a miniaturized phase fluorimeter which is combined with optical fibers using dual-lifetime referencing (DLR) as the determination method.

## Material and methods

The three aza-BODIPY dyes Cl_2_OHC_12_, apparent p*K*_a_ value of 3.93, (4-(7-(3,5-dichloro-4-hydroxyphenyl)-5,5-difluoro-1,9-diphenyl-5H-5λ^4^,6λ^4^-dipyrrolo[1,2-c:2′,1′-f][1,3,5,2]triazaborinin-3-yl)-*N*-dodecylbenzamide); ClOHC_12_, apparent p*K*_a_ value of 7.01, (4-(7-(3-chloro-4-hydroxyphenyl)-5,5-difluoro-1,9-diphenyl-5H-5λ^4^,6λ^4^-dipyrrolo[1,2-c:2′,1′-f][1,3,5,2]triazaborinin-3-yl)-*N*-dodecylbenzamide); and OHBut, apparent p*K*_a_ value of 8.90, (4-(7-(4-butoxyphenyl)-5,5-difluoro-1,9-diphenyl-5H-4λ^4^,5λ^4^-dipyrrolo[1,2-c:2′,1′-f][1,3,5,2]triazaborinin-3-yl)phenol) were synthesized in-house as described previously. The corresponding structural confirmation of these dyes is already published in literature [34]. Silanized Egyptian blue used as microcrystalline powder was prepared as described in the literature [35]. Sodium dihydrogen phosphate monohydrate was bought from Carl Roth GmbH (https://www.carlroth.com/de/de, Karlsruhe, Germany). The polyurethane hydrogel, HydroMed D4, was purchased from AdvanSource biomaterials (www.advbiomaterials.com, Wilmington, USA). 4-Dodecylbenzene sulfonic acid (DBSA), platinum(0)-1,3-divinyl-1,1,3,3-tetra-methyldisiloxane complex solution (Pt(0)-cat), and ammonium chloride were purchased from Sigma Aldrich (https://www.sigmaaldrich.com/austria.html, St. Louis, USA). Fluoropore™ membrane PTFE (0.45 μm pore size, 50 μm thickness, Teflon layer) was bought from Merck Millipore Ltd. (http://www.merckmillipore.com/AT/de, Cork, Ireland). The superphobic PES 0.2 μm membrane (no data for the layer thickness available, PES layer) was a project contribution from EMD Millipore Corporation (Billerica, MA, USA). The support, poly(ethylene naphthalate) (PEN) foil Teonex, was from Pütz (https://www.puetz-folien.com/index.php/de/, Tanusstein, Germany). Poly(dimethylsiloxane), vinyldimethylsiloxy terminated (viscosity 1000 cSt., PDMS), (25–35% methylhydrosiloxane)-dimethylsiloxane copolymer (viscosity 25–35 cSt., copolymer) and 1,3,5,7-tetravinyl-1,3,5,7-tetramethylcyclotetrasiloxane (restrainer) were purchased from abcr GmbH (https://www.abcr.de/de/, Karlsruhe, Germany). Ultrafine titan oxide (TiO_2_) P170 was from Kemira Oyj (https://www.kemira.com/; Helsinki, Finland). All chemicals and substances were used without additional purification.

Fluorescence spectra of the dyes were recorded with a Fluorolog3spectrofluorimeter (Horiba J. Y., www.horiba.com) equipped with a NIR-sensitive photomultiplier R2658 from Hamamatsu (300–1050 nm). Measurements of the DLR-referenced sensors were performed with a commercially available FirestingO_2_ reader (https://www.pyroscience.com, PyroScience GmbH, Aachen, Germany) in combination with plastic optical fibers (*Ø* 1 mm, length 1 m, https://www.ratioplast.com/index.php/en/home, Ratioplast-Optoelectronics GmbH, Lübbecke, Germany). The following settings for the calibration measurements were used: measuring time of 10 ms, LED intensity 100%, amplification of 400×, a modulation frequency of 2000 Hz, and a measuring interval of 3 s.

**Fig. 1:**
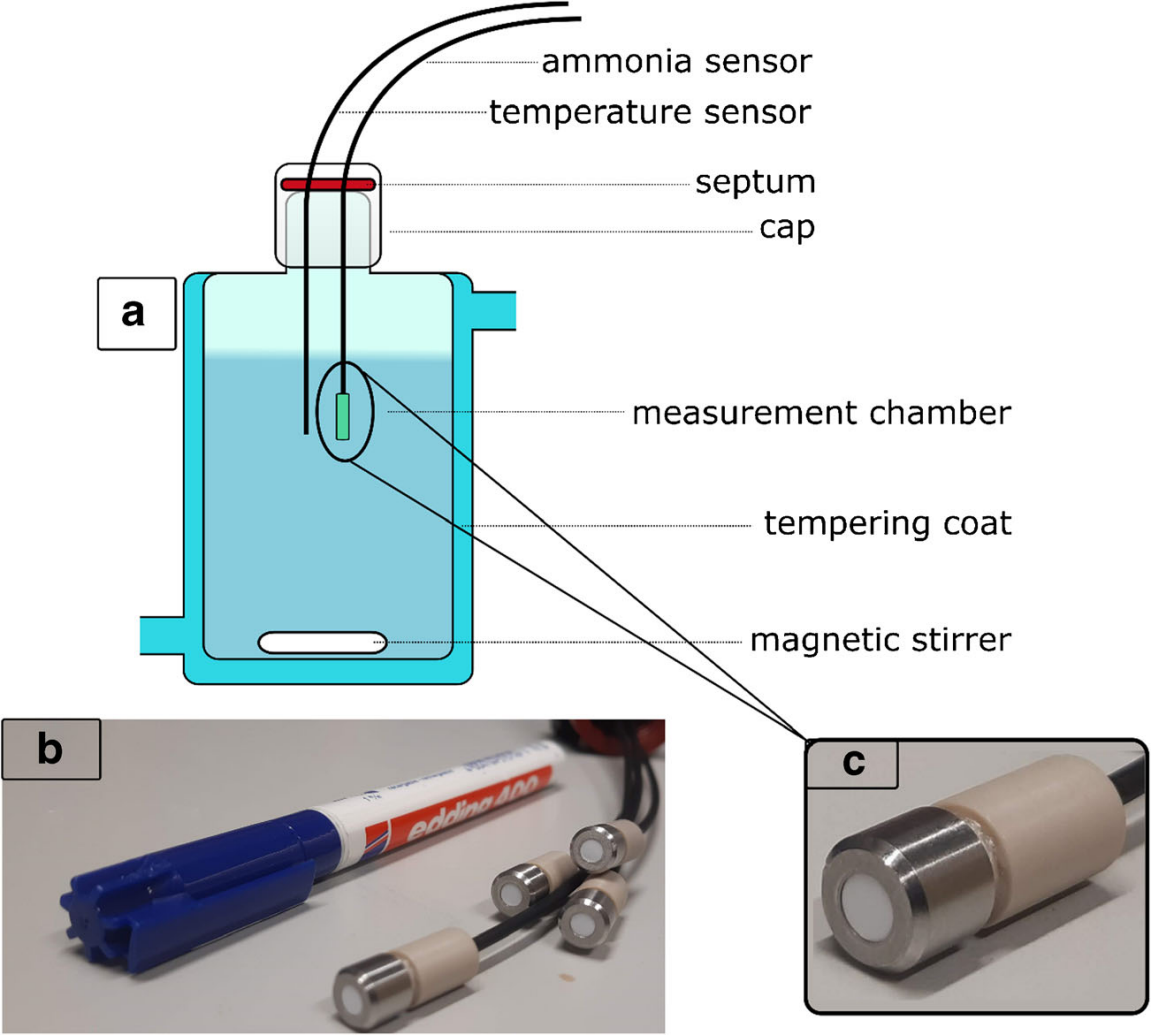
**a** Calibration measurement setup, temperature-controlled double-walled glass vessel. **b** Fiber optic sensors 5 mm spots sealed with a stainless screw cap to an optical fiber. **c** Zoomed picture of an optical sensor

All calibrations were performed in a temperature-controlled glass vessel filled with aqueous phosphate-buffered solutions at a defined pH (see Electronic Supplementary Material (ESM) Table S1) containing different amounts of free NH_3_. Additionally, a temperature sensor is implemented into the setup to the respective ammonia sensor (Fig. 1a).

### Sensor composition and preparation

We used an emulsion concept for our system based on earlier experiments. This has shown that the assembling yields in more stable sensors [36]. First, 0.25 mg (0.5 w/w) of the respective fluorescent aza-BODIPY dye is dissolved 500 mg of a hydrogel D4 (50 mg, 10 w/w) solution in EtOH/H_2_O (9 + 1). Second, acid in form of nonvolatile 4-dodecylbenzene sulfonic acid (DBSA, 1.0 mg, 100 μL of a 10 mg mL^−1^ solution in THF) is added to protonate the dye. All dye molecules are now in their “on-state” and show fluorescence. This ensures a better stabilization of the baseline signal which further yields into more reliable measurement signals. After adding the host material, that is vinyl-terminated polydimethylsiloxane (PDMS, 500 mg), the sensor “cocktail” is heated up to 100 °C to remove the solvents. Thereby, it is emulsified for 20 min (2000 rpm). After cooling to room temperature, (25–35% methylhydrosiloxane)-dimethylsiloxane (copolymer, 20 μL), 1,3,5,7-tetravinyl-1,3,5,7-tetramethylcyclotetrasiloxane (restrainer, 20 μL of a 10-mg mL^−1^ stock solution in cyclohexane) and silanized Egyptian blue as a reference material (5 mg) are added to the emulsion. Before adding platinum(0)-1,3-divinyl-1,1,3,3-tetra-methyldisiloxane complex solution (Pt(0)-cat, 10 μL), the “cocktail” is emulsified at room temperature for 5 min The emulsion is knife-coated (25 μm wet film) on a dust-free PEN support (125 μm). Finally, a hydrophobic layer (either a Fluoropore membrane PTFE (A1, B1, and C1) or a hydrophobic PES layer (A2, B2, and C2) is laid carefully onto the wet film. In the case of sensor B3, a mixture of PDMS and TiO_2_ is used as an ion barrier. A stock solution of 5 g PDMS, 1.0 g TiO_2_, and 200 μL copolymer in 2 mL cyclohexane is used. The respective amounts of restrainer and catalyst are added before knife coating (25 μm wet film onto the sensing layer).

After 4 h, the PDMS is polymerized and the sensor material is ready to use. Sensor spots are punched out with a stainless steel ferrule (*Ø* 5 mm) and fixed via a stainless steel screw cap to an optical fiber (see Fig. 1b, c). In Table 1, all sensors classified by indicator dye and ion barrier are listed.

**Table 1:** Sensor composition listed by used dye and ion barrier

Sensor	Dye	Ion barrier
A1	Cl_2_OHC_12_	Teflon
A2	Cl_2_OHC_12_	hydrophobic PES
B1	ClOHC_12_	Teflon
B2	ClOHC_12_	hydrophobic PES
B3	ClOHC_12_	TiO_2_/PDMS
C1	OHBut	Teflon
C2	OHBut	Hydrophobic PES

### Dual-lifetime referencing

Fluorescence intensity measurements are influenced by many factors like fluctuation of the excitation source, performance of the detector, and electronic interferences. Due to this, we have chosen a different sensing method. It is called dual-lifetime referencing (DLR) whereby the fluorescence intensity ratio of an analyte-sensitive dye with a short lifetime and an inert reference having a long lifetime is contributing to an overall phase shift (dphi). Therefore, both are excited simultaneously with a modulated light pulse. Further conversion of dphi into cotangent values represent a direct proportionality to the fluorescence intensity [37]. As reference, the indicator Egyptian blue was chosen due to its photochemical inert behavior and spectral compatibility to the aza-BODIPY dyes. Both indicators can be excited with a red LED, which makes them compatible with the commercially available FirestingO_2_ reader from Pyroscience, and emit in the NIR spectral region.

## Results and discussion

### Choice of materials

**Fig. 2:**
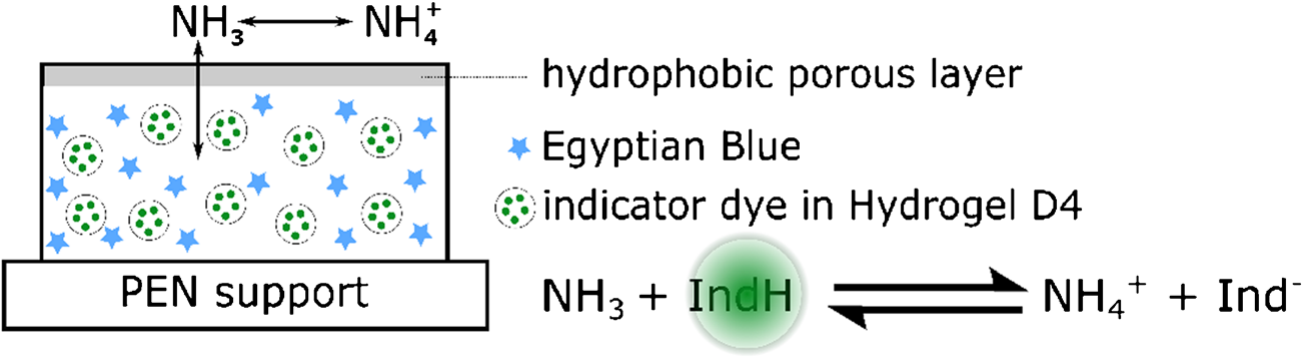
Sensor concept: gaseous NH_3_ diffuses through a hydrophobic layer into the sensing layer to the dye. Thereby, it deprotonates the hydroxy group of the dye and shuts off the fluorescence

A common concept in sensor technology is the layer by layer concept whereby the sensing layer is knife coated on top of a support material. On top of the sensing layer, either an optical isolation or another type of protection layer (e.g., ion barrier) is built. Previous studies have shown that the layer by layer concept has no long-term stability for this application; we have chosen an emulsion concept (Fig. 2) [36]. The study by Strobl et al. has overcome the problem of poor sensor stability but did not yield into a quick sensor response. Since we wanted to build a modular toolbox for ammonia sensing suitable for a wide range of applications, we improved this sensor concept. Therefore, three aza-BODIPY dyes (different in their apparent p*K*_a_ values) as indicators, a PDMS matrix as a host material, a hydrogel D4 for dye incorporation, and three different ion barriers were tested (see Table 1).

**Fig. 3:**
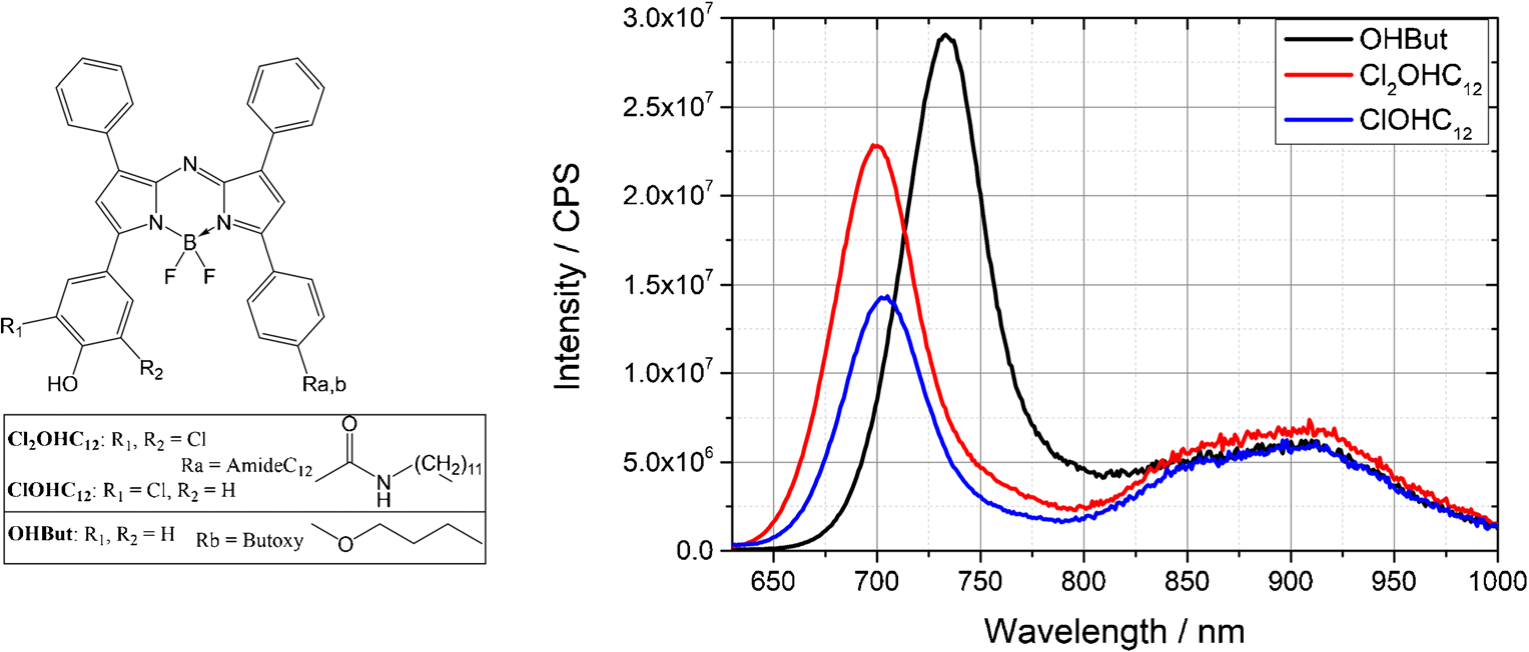
Left: Molecular structure of three aza-BODIPY dyes: Cl_2_OH_12_, ClOHC_12_, OHBut; right: emission spectra (λ_exc = 610 nm) of sensor foils with either Cl_2_OHC_12_ (red), ClOHC_12_ (blue), or OHBut (black) as an indicator in the protonated state and Egyptian blue (λ_max = 900 nm) as a reference

The dynamic range of the sensor is limited to the apparent p*K*_a_ of the indicator dye, since this sensing principle is set upon an acid-base system (Fig. 2). The lower the p*K*_a_ value, the more sensitive the sensor is and vice versa. As a basic structure, we have chosen aza-BODIPY dyes (see Fig. 3, left) due to their high photostability, spectral properties (excitable via red LED, emit in the NIR region (Fig. 3, right), good brightness), and structural diversity (see Fig. 3, left) [31]. By introducing electron-withdrawing or electron-donating groups in the two ortho-positions to the phenol group, the apparent p*K*_a_, which was measured in a solution of EtOH/H_2_O 1 + 1, of the hydroxyl group can be varied from 3.9 up to 8.9 [34]. The fact that we can use different indicator dyes in our system yields into a broad range of possible application. On the one hand, we can build a trace sensor which is suitable for NH_3_ concentrations down to low micrograms per liter. These are demanded for fish farming. On the other hand, we can also monitor ammonia within biocatalytic reactions in which common concentrations up to several milligrams per liter of free NH_3_ can be expected. In its protonated state, the dye emits fluorescence (IndH), whereas the proton is transferred in the presence of ammonia and the fluorescence is switched off due to a photoinduced electron transfer (PET; see Fig. 2).

For a rapid sensor response, a fast diffusion of ammonia through the ion barrier and the host matrix is favorable. As host material PDMS, which acts permeation-selectively for hydrophobic species, is tested due to its outstanding high diffusion rate for ammonia [38]. To avoid interferences caused by hydrophilic species, this sensor concept requires a ion barrier on top of the sensing layer. In this study, three different materials were tested as possible ion barriers. First, the hydrophobic Teflon membrane filter is known to have a high diffusion rate for ammonia due to its highly porous structure as well as its thin layer thickness. It is therefore suitable as a protection layer. Due to its hydrophobic nature, cross-sensitivity towards varying salinity is also negligible [39]. Additionally, fluoropolymers are highly resistant to biofouling which makes them suitable for measuring in environmental samples or bioreactors (see Fig. 1b, c, white 5 mm spot) [40]. Second, a mixture of PDMS/TiO_2_ was knife coated on top of the sensing layer. Thereby, the PDMS works as an ion barrier and the TiO_2_ acts as an optical isolation. As a third material, a hydrophobic PES layer was laid on top of the wet sensing layer. This layer might be an alternative to the mechanically more fragile Teflon layer.

### Sensing performance and stability

**Fig. 4:**
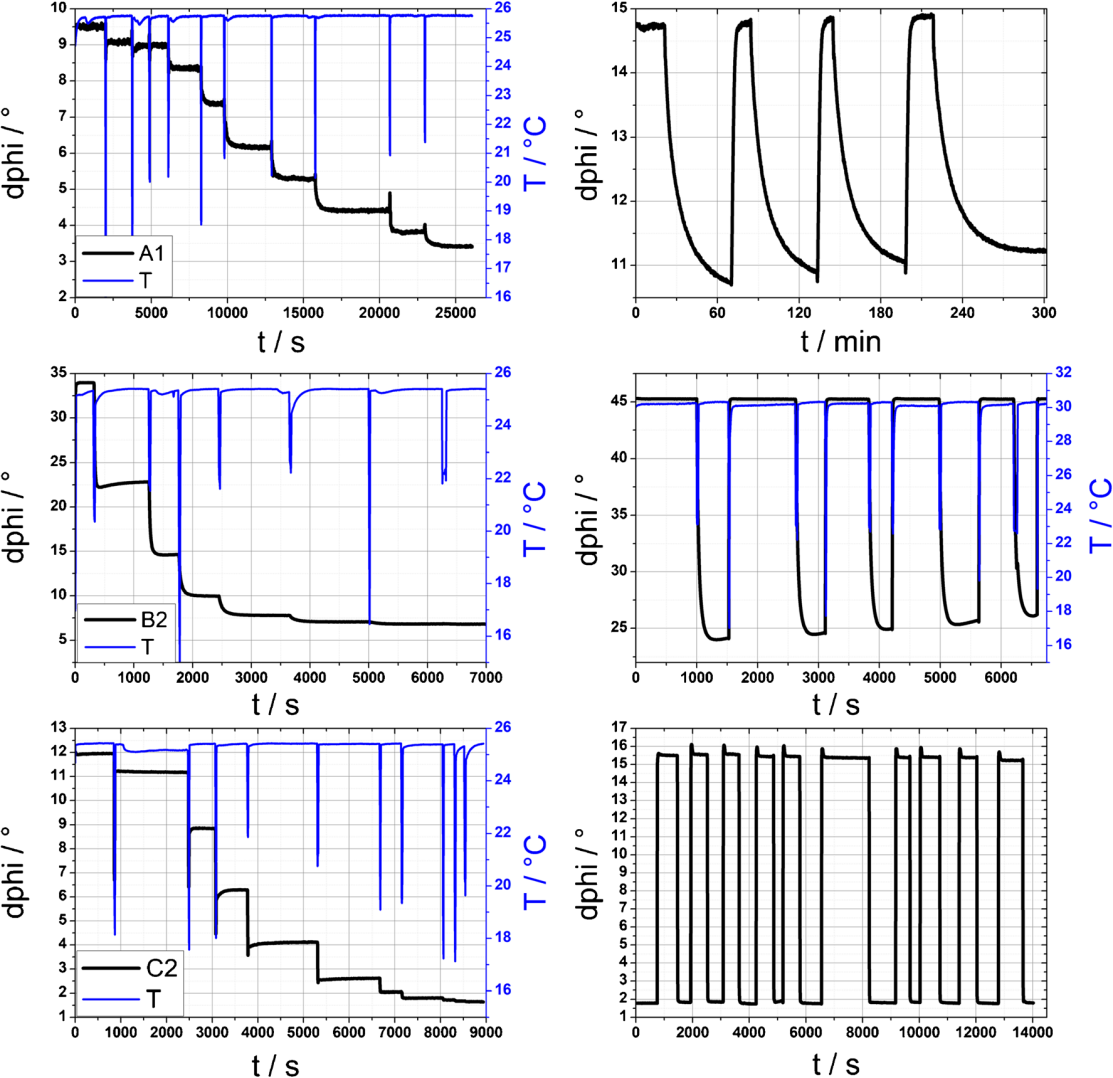
Left: response curves of sensor A1, B2, and C2, the respective calibration concentrations can be seen in Fig. 5; right: hysteresis experiments of sensor A1 (10 mg L^−1^ NH_3_ vs 0 buffer), B2 (100 mg L^−1^ vs 10 mg L^−1^ NH_3_), and C2 (6 g L^−1^ NH_3_ vs 0 buffer)

In its left part, Fig. 4 shows the sensor response of the three sensors A1, B2, and C2 whereby a decrease of the phase angle dphi correlates to an increase in the concentration of the protonated dye form. This figure also shows the reversibility of the sensors. Due to their high stability and good reversibility combined with the right proton barrier, these sensors are the best candidates for the specific application fields: A1 as sensor for fish farming or environmental monitoring (measurement range for free ammonia in the low μg L^−1^ area), B2 in biocatalysis (measurement range for free ammonia up to more than 10 mg L^−1^), and C2 in chemical reaction monitoring (measurement range for free ammonia more than 1 g L^−1^). Figure S4 (see the ESM) shows the response curves and stability tests of the remaining sensors (A2, B1, B3, and C1).

**Fig. 5:**
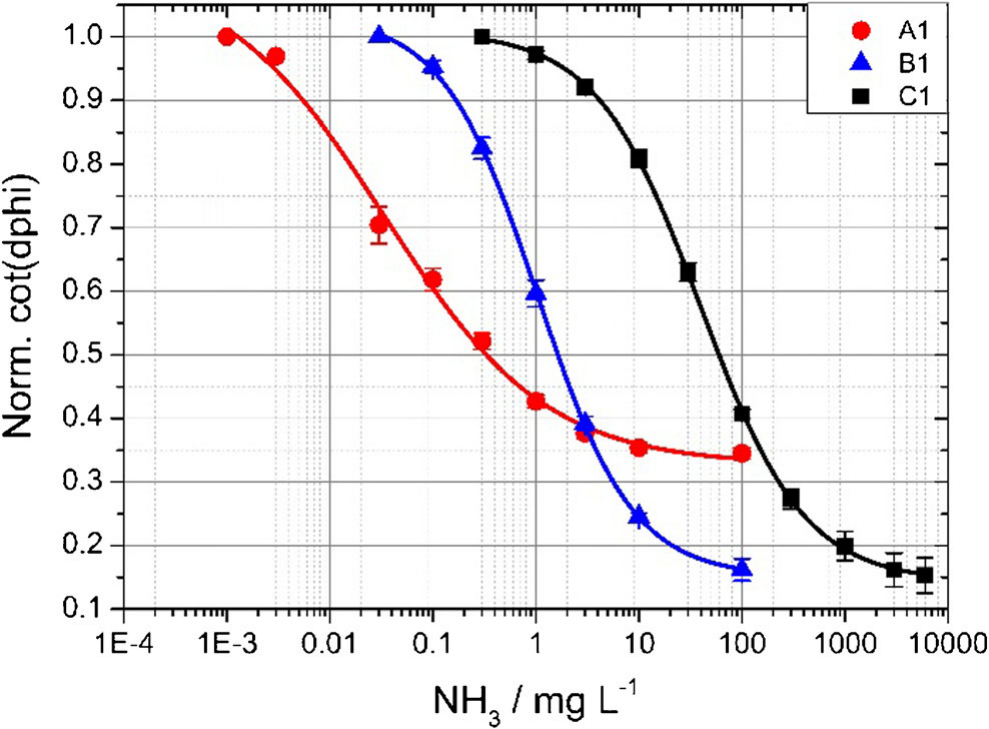
Calibration curves of the three different ammonia sensors at 25 °C, using ion barrier porous Teflon membrane

Plotting the cot(dphi) against the NH_3_ concentration yields a sigmoidal curve (Fig. 5) which is further fitted by the following Boltzmann fit (1):

1 $$ y=\mathrm{A}2+\frac{\mathrm{A}1-\mathrm{A}2}{1+{10}^{\frac{\log (x)-{x}_0}{dx}}} $$

Here, *y* is the value of the normalized cot(dphi), A1 and A2 represent the upper and the lower plateau, *x* represents the concentration of the calibration point, *x*_0_ is the point of inflection, and *dx* is the slope of the curve.

Figure 5 shows the calibration curves of three different sensors (A1, B1, C1). They vary in their indicator dye but all have the same ion barrier (see Table 1). A1 (red line) uses the dye with the lowest apparent p*K*_a_ of 3.93 value, whereas C1 (black line) incorporates the dye with the highest apparent p*K*_a_ of 8.90. To summarize, the lower the hydroxy groups p*K*_a_ of the pH dye, the more sensitive the sensor. The flexible tuning of the dynamic range of the sensor offers a big variety in many different application fields. Since the crucial ammonia concentration for many living organism is 25 μg L^−1^, sensor type A (LOD of 0.23 μg L^−1^) has high potential for application in environmental monitoring [5]. Sensor type B (apparent p*K*_a_ of 7.01, LOD of 28 μg L^−1^) has its dynamic range between approximately 1 and 100 mmol L^−1^ TAC (total ammonia concentration, NH_3_ + NH_4_^+^) which is suitable for monitoring biocatalytic reactions or fermentation processes [41–43]. Sensor type C (LOD of 0.51 mg L^−1^) can even measure higher concentrations of ammonia that can be interesting for industrial use or in flow chemistry for reaction monitoring. There, higher amounts of ammonia can be dissolved in water due to the high pressure in flow chemistry. The calculation of the LOD can be found in the ESM.

**Fig. 6:**
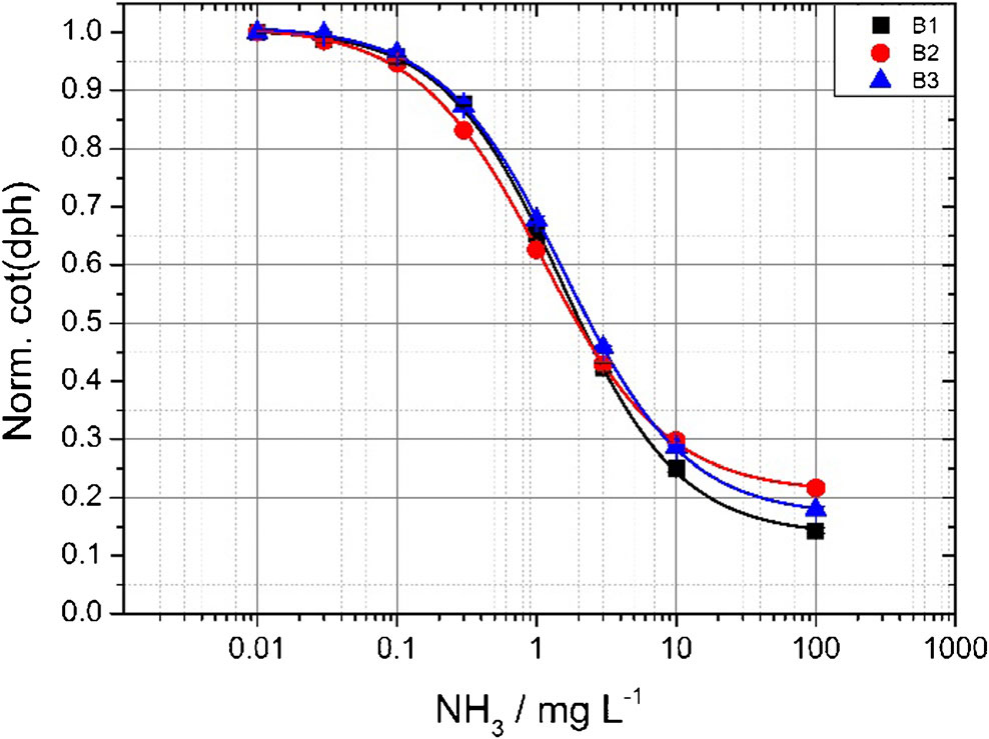
Calibration curves of the three different ion barriers at 25 °C, using dye ClOHC_12_

In the following section, one sensor type is always used to explain the results in detail. The trends in pH, temperature influence, and response times for different protection layers are the same. We have tested three different ion barriers in this study. We wanted to investigate the influence of the ion barrier on the sensing properties of the various sensors. Therefore, we calibrated sensors B1, B2, and B3 at the same time in the same buffer solutions. This experiment was performed for all sensor types and yields to the same trend in their results. Figure 6 shows that there are hardly any differences in lower concentration ranges. However, a small difference is detectable at higher concentrations. This might be attributed to inhomogeneities of the sensing layer that might be created within the emulsifying process. Nevertheless, in all three cases, the sensor works reversibly and with stability. Additionally, all three protection layers were tested against proton impermeability by dipping them into buffered solutions with different pH values (see ESM Figs. S5, S6, and S7). All three measurements show hardly any difference in the signals when changing the buffers, even though some of the pH buffers are far beyond the apparent p*K*_a_ value of the used aza-BODIPY dye. This correlates with former studies [36, 39, 44]. Hysteresis experiments were performed for stability tests. The best results show sensor C2 (Fig. 4, right part). This sensor outperforms all others and shows barely any drift after 10 cycles—neither at very high free ammonia concentrations (6 g L^−1^) nor at 0 buffer. The sensor response is still fast and the signal stable after reaching the respective plateau. Other sensor types do show a less stable performance within this experiment. Especially when changing from very high ammonia concentrations to 0 buffer, their baseline signal rises over time. This can be explained with a slower mass transfer of ammonia between the hydrogel and the buffer solution. This effect is more pronounced in the trace measurements than at very high concentrations. Further information about the long-term stability of the sensors can be found in the ESM (Fig. S8).

**Fig. 7:**
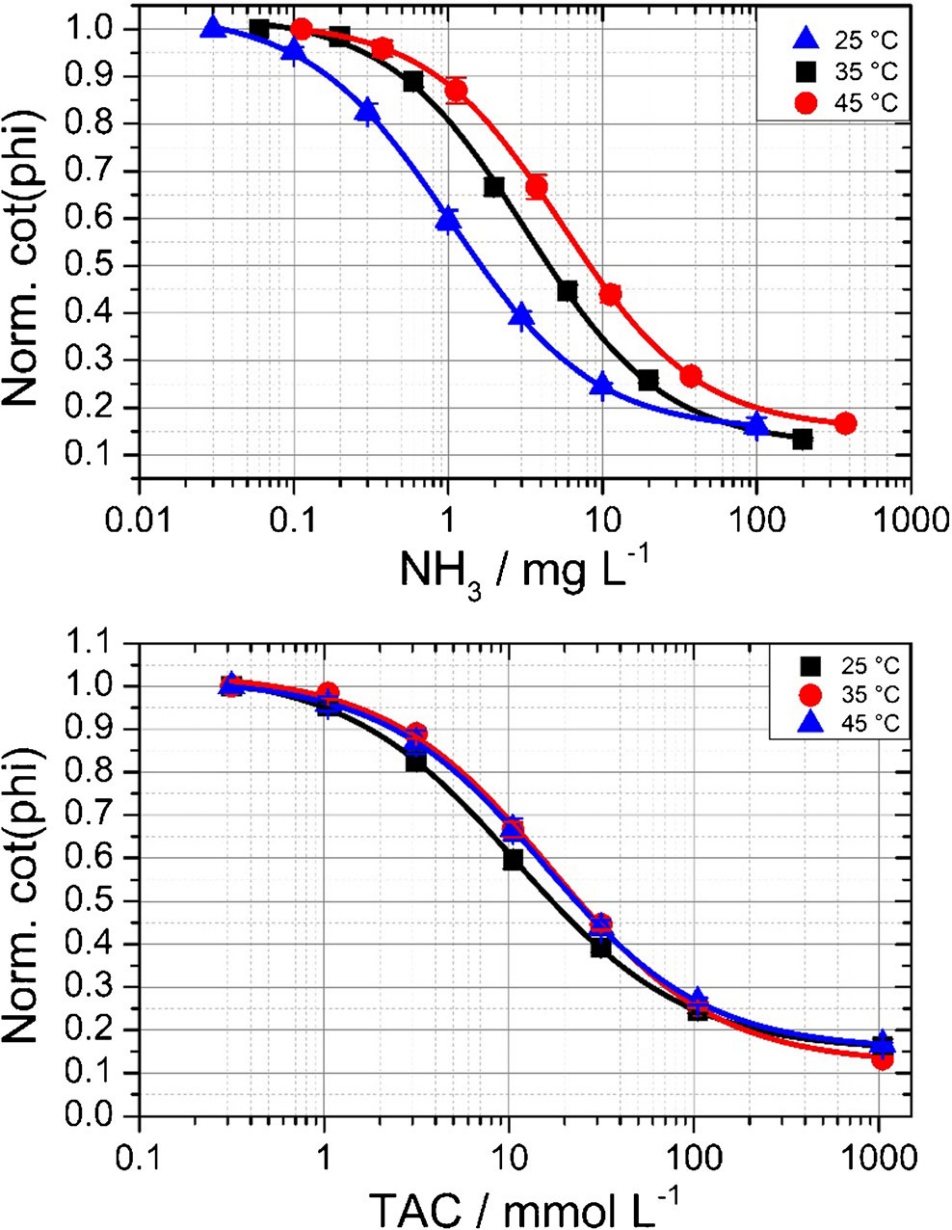
Influence of temperature, sensor B1, upper part plotted vs free NH_3_ in mg L^−1^, lower part plotted vs TAC in mmol L^−1^

The temperature dependency is shown in Fig. 7. For sensor type B, we estimated that temperatures between 25 and 45 °C would be reasonable since this sensor is suitable for operations in biotechnology. Plotting the free ammonia concentration against the normalized cot(dphi) measured at different temperatures can yield to a wrong interpretation of the results. The upper graphic in Fig. 7 shows a shift of the calibration curve at different temperatures. When increasing the temperature, the sensor seems to get less sensitive but we have to consider that the ammonia ammonium equilibrium strongly depends on the temperature and the pH (see ESM Figs. S1 and S2). Based on this information, we only see the shift in free ammonia concentration of each buffer at different temperatures and not a different sensor behavior. For the correct evaluation of the data, plotting of normalized cot(dphi) against TAC is useful. The lower graphic in Fig. 7 shows three calibration curves of sensor B1 at 25, 35, and 45 °C. The temperature influence on the sensing properties of the sensor between 35 and 45 °C is negligibly low, whereas at 25 °C a small shift can be detected.

Besides different temperatures, the response time of the sensor system is basically limited to two main factors. The diffusion rate of ammonia through the PDMS matrix to the dye molecules, which are incorporated in the hydrogel, and the different ion barriers having an influence on the response time. Since the PDMS matrix is used in all three sensor types, we focus on the different protection layers. While the sensors of categories A, B, and C vary in their indicator dye and therefore in their dynamic range due to different p*K*_a_ values, we compare response and recovery times (*t*_90_) within one category. As examples, sensors B1–B3 measured at 25 °C are used for comparing all three protection layers. Concerning their response as well as their recovery times, sensors B1 and B2 are similar. At the lowest calibration point (0.01 mg L^−1^), B1 has a response time of 10 min, whereas B2 is slightly faster with 9 min. The recovery times behave similarly. In contrast to that, B3 is clearly slower than the other two (*t*_90_ = 55 min). The same trend can be seen at the highest calibration solution (100 mg L^−1^). There, B1 responds within 15 s and B2 in 25 s, whereas B3 requires 390 s. An overview of the response and recovery times of the other sensors is given in the ESM (Table S2).

## Conclusion

To summarize, we were able to develop a variety of optical sensors for application in analytical ammonia determination. Therefore, we implemented the respective fluorescent and photostable aza-BODIPY dye in hydrogel D4 which is emulsified into a PDMS matrix. Varying p*K*_a_ values of the dye’s hydroxyl group yield to different dynamic ranges of the respective sensor making this system a powerful and flexible tool for ammonia determination in totally different fields. Possible applications range from environmental monitoring (fish farming; sensor type A) to biotechnological applications (bioreactor, biocatalysis; sensor type B) and even flow chemistry (reaction monitoring, sensor type C). Compared to our previous study published by Strobl in 2017 in this field, we decreased the response time of the trace sensor (type A, 10 mg L^−1^–1 μg L^−1^) drastically and expanded the possible application fields by introducing two new sensor types (B and C). Thereby, sensor type B covers the concentration range of free dissolved ammonia from 100 mg L^−1^ down to 0.1 mg L^−1^ and type C is suitable for higher concentrations from 1 g L^−1^ to 1 mg L^−1^. The decrease in sensor response time can be attributed to matrix changes and the usage of different protection layers. Studying various ion barriers yield to significant differences in the response time of the sensor. Here, hydrophobic PES and the porous Teflon layer yield to comparable results and clearly outperform a knife-coated PDMS/TiO_2_ layer. No significant influence of pH can be observed.

## Electronic supplementary material

ESM 1 (PDF 1068 kb)
