# Supplementary material for: Optical ammonia sensors based on fluorescent aza-BODIPY dyes— a flexible toolbox
Source: Anal Bioanal Chem. 2020 Aug 24;412(27):7559–67. doi: 10.1007/s00216-020-02891-3 (PMC7533255; doi:10.1007/s00216-020-02891-3)
Supplement: Supplementary file 1 — (PDF 1068 kb) [file 216_2020_2891_MOESM1_ESM.pdf]

Analytical and Bioanalytical Chemistry

Electronic Supplementary Material

Optical ammonia sensors based on fluorescent aza-BODIPY dyes – a flexible toolbox

Maximilian Maierhofer, Veronika Rieger, Torsten Mayr

Equilibrium of NH_3 to NH_4^+ strongly depends on temperature and pH

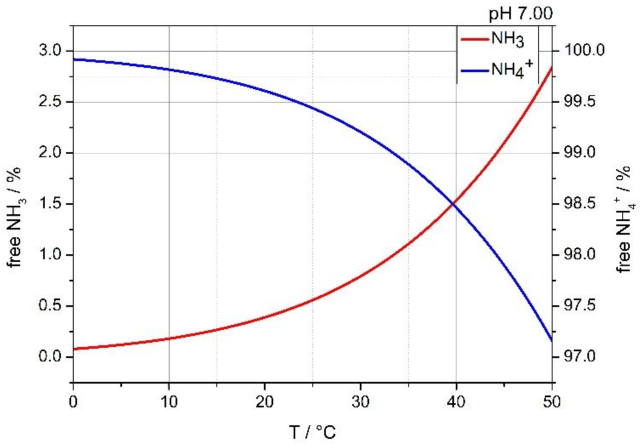

Fig. S1 Temperature dependency of NH_3 to NH_4^+ at pH 7.00

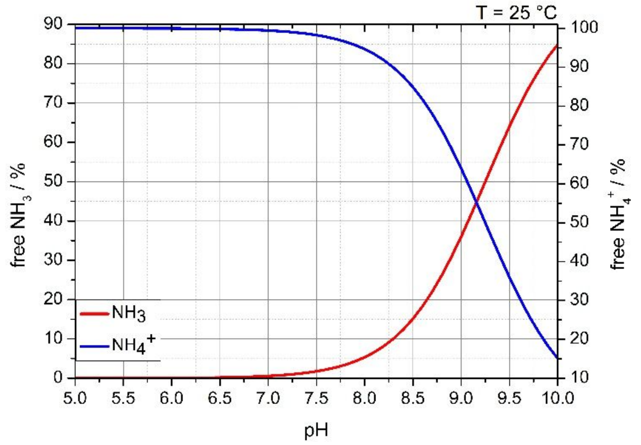

Fig. S2 pH dependency of NH_3 to NH_4^+ at T=25 °C

Material and methods

For pH cross sensitivity experiments we used a standard 20 mM universal buffer (citrate, CAPS, BIS-TRIS and TRIS). Thereby the pH was adjusted with 1 M HCl or NaOH. Read out was performed with a digital pH meter (Seven Easy, Mettler Toledo, www.mt.com) which was calibrated at 25 °C with standard buffers of pH 7.0, 4.0 and 10.0 (WTW, www.wtw.com).

Buffer calculation

100 mM phosphate buffer solutions (pH 7.00 and pH 8.00) were prepared and an equivalent amount of ammonium chloride, was dissolved in each buffer. The concentration of free ammonia was calculated by the Henderson-Hasselbalch equation:

$$dG^0 = R * T * \ln(k)\,; dG = 52808; R = 8.3144$$

$$pK_a = \frac{dG^0}{\ln(k) * R * T}$$

$$pH = pK_a + log_{10}\left(\frac{[NH_3]}{[NH_4^+]}\right)$$

free NH_3:

$$[NH_3] = \frac{10^{pH-pK_a}}{10^{pH-pK_a} + 1}$$

Example:

$$\frac{free\ NH_3 \left[\frac{\mu g}{L}\right]}{M(NH_3)} = free\ NH_3 \left[\frac{\mu mol}{L}\right]$$

$$\frac{free\ NH_3 \left[\frac{\mu mol}{L}\right]}{Anteil\ [NH_3] * 1000} = TAC\ \left[\frac{mmol}{L}\right]$$

$$NH_4Cl\ [mg] = \frac{TAC\ \left[\frac{mmol}{L}\right] * M[NH_4Cl] * V\ Flasche\ [mL]}{1000}$$

Calibration experiments were performed by placing 4 optical fibers in a temperature controlled glass vessel (Figure 1) filled with the respective buffer solution. The fibers were connected to a compact phase fluorimeter from Pyroscience (Fig. S3).

Limit of detection

Limit of detection (LOD) is determined to validate the sensitivity of the sensors. LOD is the lowest concentration of the analyte that can be detected. It is calculated with the following formula.

$$LOD = 3.3 * SD$$

$$SD\ \ldots standard\ deviation\ of\ the\ blank, phosphate\ buffer\ without\ ammonia$$

Table S1 Buffer solutions with different ammonia concentration for sensor calibration

Cl_2OHC_{12}	$ClOHC_{12}$	OHBut
free NH_3 [mg L^{-1}] at 25 °C, pH 7.00		free NH_3 [g L^{-1}] at 25 °C, pH 8.00
100	100	6
10	10	3
3	3	1
1	1	0.3
0.3	0.3	0.1
0.1	0.1	0.03
0.03	0.03	0.01
0.01	0.01	0.003
0.003	-	0.001
0.001	-	0.0003

Measurement setup:

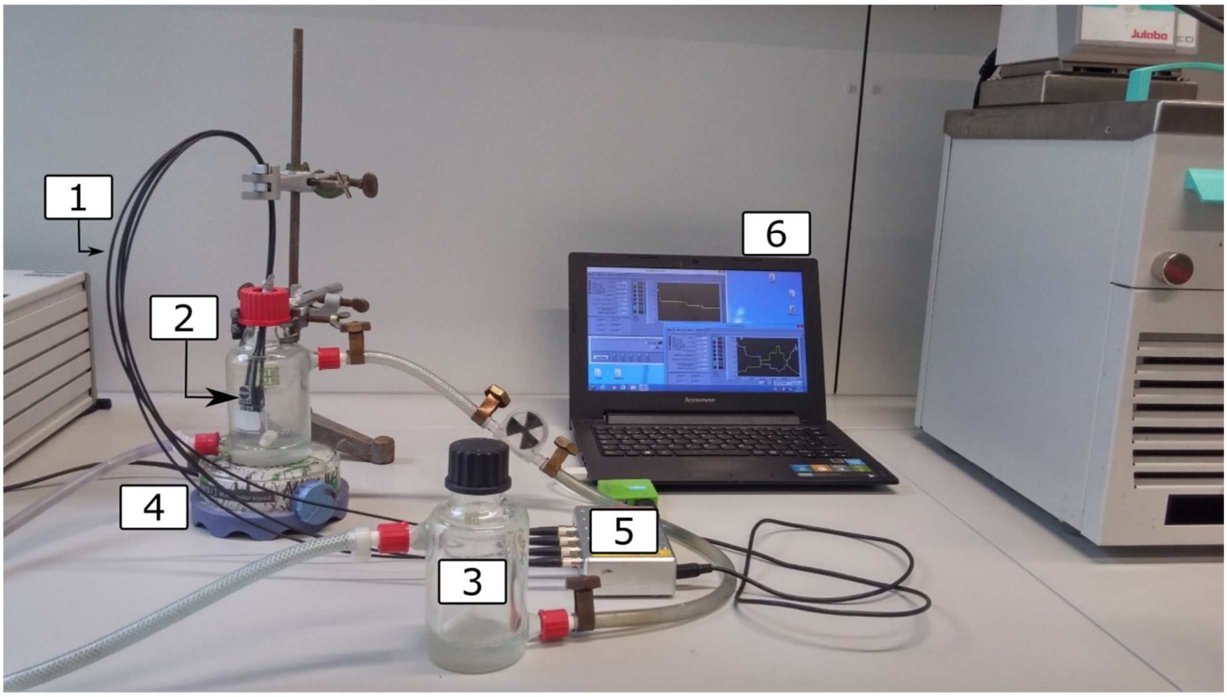

Fig. S3 measurement setup: optical fibers (1), optical sensors (2), temperature-controlled glass vessel as measuring chamber (3), stirrer (4), compact phase fluorimeter (5), laptop (6)

Response and recovery times t_{90}:

Table S2 Response and recovery times t_{90} of all sensors at the lowest and the highest calibration point at 25 °C

**-**	**Teflon**	**PES**	**Teflon**	**PES**	**PDMS TiO_2**	**Teflon**	**PES**
**Sensor**	**A1**	**A2**	**B1**	**B2**	**B3**	**C1**	**C2**
**Lowest calibration point**	100 min<br>100 min	60 min<br>70 min	10 min<br>10 min	9 min<br>9.5 min	55 min<br>60 min	150 s<br>180 s	42 s<br>42 s
**highest calibration point**	30 s<br>60 s	60 s<br>90 s	15 s<br>30 s	25 s<br>30 s	6.5 min<br>10 min	12 s<br>18 s	9 s<br>15 s

Response curves and hysteresis testing:

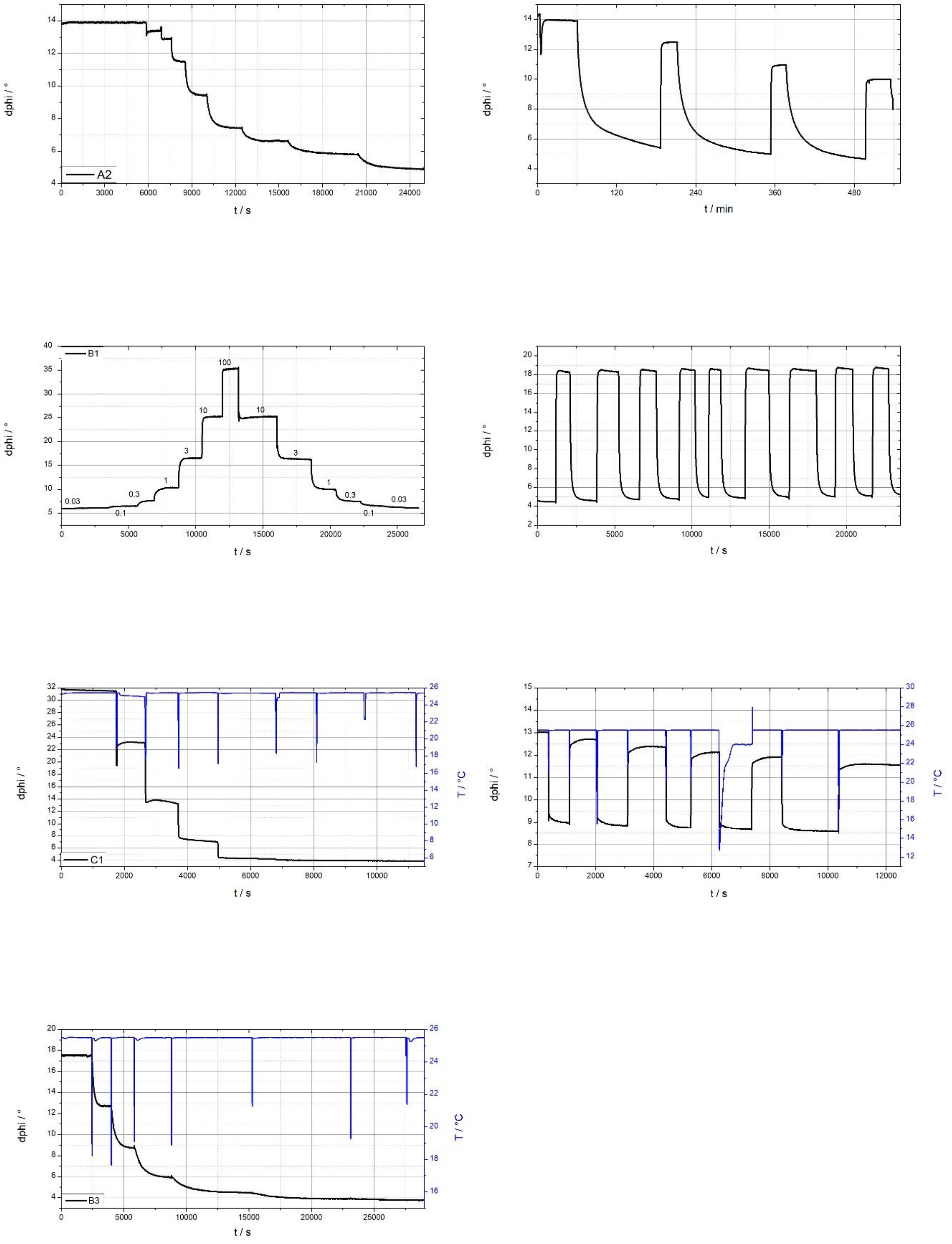

Fig. S4 left: response curves of sensor A2, B1, C1 and B3; right: hysteresis experiments of sensor A2 (1 mg L^{-1} NH_3 vs 0 buffer), B1 (100 mg L^{-1} NH_3 vs 0 buffer) and C1 (300 mg L^{-1} NH_3 vs 100 mg L^{-1} NH_3)

pH influence on Teflon layered sensor

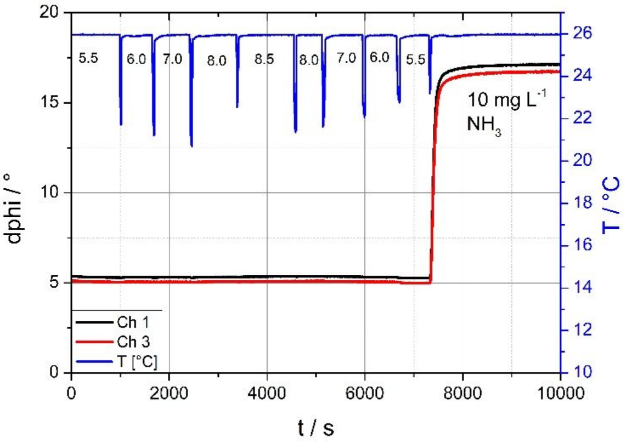

Fig. S5 Influence of pH on the sensor performance, ion barrier porous Teflon layer

pH influence on PDMS/TiO2

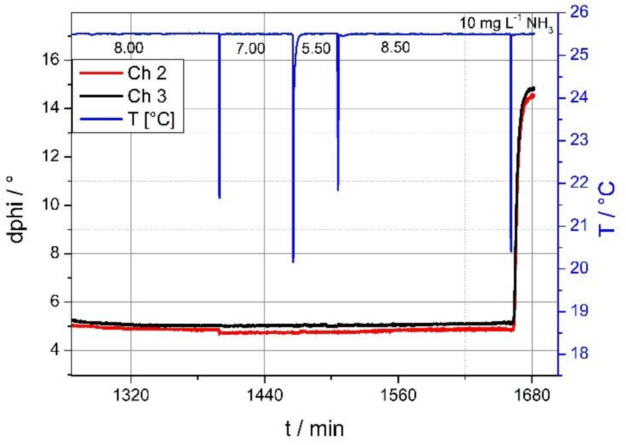

Fig. S6 Influence of pH on the sensor performance, ion barrier PDMS/TiO_2 layer

pH influence on hydrophobic PES layer

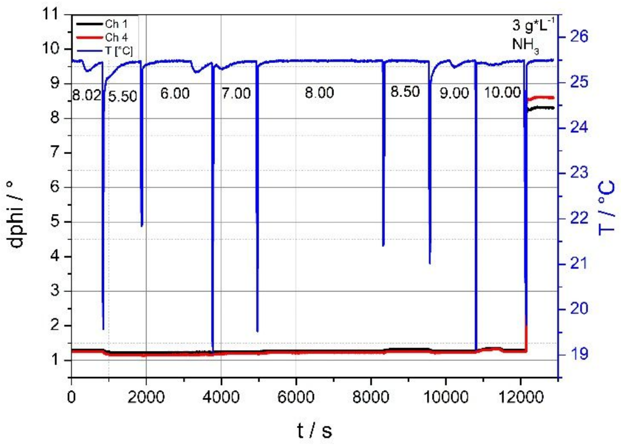

Fig. S7 Influence of pH on the sensor performance, ion barrier hydrophobic PES

Long-term stability:

First, new sensors have been calibrated as mentioned previously (straight fits). After this, they have been stored in a phosphate buffer at pH 7.00 with 100 mg L^{-1} of free NH_3 for two weeks. Then again three calibrations at different temperatures have been performed with the sensors (dotted fits). The result shows a measurable difference at 25°C but hardly any differences at higher temperatures.

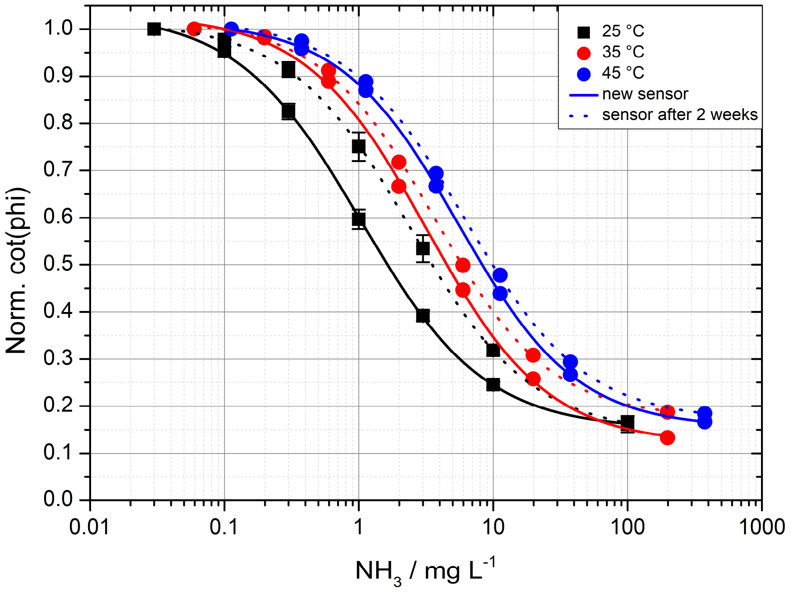

Fig. S8 Long-term stability of the sensors. new sensors were calibrated (straight fit), then stored in 100 mg L^{-1} NH_3 phosphate buffer for two weeks and again calibrated (dotted fit)
